# Morphometric and histomorphometric evaluations of high-purity macro/microporous beta-tricalcium phosphate in maxillary sinus floor elevation: preliminary results on a retrospective, multi-center, observational study

**DOI:** 10.1186/s12903-021-01797-5

**Published:** 2021-09-16

**Authors:** Atsushi Fujita, Chonji Fukumoto, Tomonori Hasegawa, Yuta Sawatani, Hitoshi Kawamata

**Affiliations:** Department of Oral and Maxillofacial Surgery, Dokkyo Medical School of Medicine, 880 Kita-Kobayashi, Shimo-Tsuga, Mibu, Tochigi 321-0293 Japan

**Keywords:** Augmentation, High-purity macro/microporous β-TCP, Maxillary sinus floor elevation

## Abstract

**Background:**

The present study examined the effectiveness of high-purity macro/microporous beta-tricalcium phosphate (HPMM β-TCP) as a bone grafting material for maxillary sinus floor elevation by morphometric, histopathological, and histomorphometric evaluations.

**Methods:**

Ten unilateral maxillary sinus floor elevation procedures using 100% HPMM β-TCP were performed in 10 patients. Morphometric evaluation was carried out by computed tomography (CT) imaging immediately after augmentation and prior to dental implant placement 7 months later. Histopathological and histomorphometric evaluations were carried out by bone biopsy retrieval at the time of dental implant placement 7 months after sinus floor elevation.

**Results:**

All 10 sinus floor elevations were successful. Morphometric evaluation by CT showed that the vertical height and volume gained by sinus floor elevation decreased 7 months after surgery. Histopathological evaluation of bone biopsy retrieval specimens showed no signs of inflammation at the newly formed bone area and the native alveolar bone area. New bone formation was observed at the cranial side from the native alveolar bone. The newly formed bone had a trabecular structure and was in intimate contact with the HPMM β-TCP material. Histomorphometric evaluation of bone biopsy retrieval specimens showed an average new bone volume of 33.97% ± 2.79% and an average residual HPMM β-TCP volume of 15.81% ± 4.52%.

**Conclusions:**

In this study, HPMM β-TCP showed osteoconductive properties for vertical augmentation of the atrophied maxilla by means of a maxillary sinus floor elevation procedure allowing subsequent dental implant placement after a 7-month healing period.

## Background

Dental implant placement in the posterior maxilla often requires surgical intervention on the maxillary sinus floor area because of insufficient bone volume. Maxillary sinus floor elevation performed for placing dental implants in this region was first reported by Boyne et al. [1] and Tatum [2]. This surgical procedure makes the dental implant placement possible by increasing the alveolar bone height in this region. Various graft materials have been used to elevate the maxillary sinus floor. Autogenous bone grafts are considered to be the gold standard, because they are not immunogenic and they have osteogenic, osteoinductive, and osteoconductive properties [1, 3]. However, there are several disadvantages, including postoperative complications at the donor site, such as limping when the graft is taken from the iliac crest, prolonged healing time at the donor site, requirement for general anesthesia and hospitalization, increased cost of treatment, and unpredictable resorption of the new bone by grafting [4, 5]. These disadvantages have led to a search for eligible graft materials that are a biocompatible and osteoinductive or at least an osteoconductive alternative to autogenous bone substitutes in sinus floor elevation.

Various bone-grafting materials such as xenografts (bovine or coralline hydroxyapatite) [6–9], allografts (freeze-dried demineralized bone) [10], and alloplasts, such as hydroxyapatite, beta-tricalcium phosphate (β-TCP), bioactive glass [11–15], are presently being used as alternatives or supplements to autogenous bone. These biomaterials act as a scaffold for bone formation [16].

Anorganic bovine-derived hydroxyapatite (BHA), one of the non-resorbable xenografts, has been shown to be a safe and biocompatible bone graft material with osteoconductive properties [17]. In addition, several experimental and clinical studies have shown successful results with BHA graft materials when used for maxillary sinus floor elevation [6–9, 16]. BHA, prepared from Australian cattle, is processed chemically and through heat treatments to remove proteins and other organic substances to ensure the product has no prion and is, thus, non-antigenic [18]. The resulting particles are similar to human bone particles with regard to physical and chemical composition, ensuring the graft provides a conductive scaffold for bone formation.

β-TCP, a ceramic alloplast, is a common graft material with osteoconductive properties that has shown promising results [19, 20]. Several authors have reported β-TCP as a satisfactory graft material for maxillary sinus floor elevation [16–22]. The biomaterial is replaced by newly formed bone [21, 22]. Recently, high-purity macro/microporous β-TCP (HPMM β-TCP: Arrowbone-β-dental®, brainbase©, Tokyo, Japan) was released as a resorbable bone substitute [23].

The purpose of this preliminary, retrospective, multi-center, observational study was to evaluate the quality and quantity of bone formation in a maxillary sinus floor elevation procedure using only HPMM β-TCP with a 7-month healing period. Clinical, radiological, morphometric, and histomorphometric parameters were examined.

## Methods

### Patients

The study consisted of 10 (3 male, 7 female) patients who were partially edentulous in the posterior maxilla and required dental implant placement in two private dental clinics and Department of Oral and Maxillofacial Surgery, Dokkyo Medical School of Medicine from June 2017 to November 2018. The mean age of the patients was 57.2 ± 2.43 years (range, 41–67 years). The inclusion criteria were a residual alveolar ridge height of < 5 mm and a width of ≥ 5 mm. Panoramic radiographs and computed tomography (CT) images were taken, and these were used to evaluate the residual alveolar ridge height. Maxillary sinus floor elevation followed by dental implant placement was planned. The exclusion criteria were unsatisfactory oral hygiene (plaque control score > 20%) [24], acute maxillary sinusitis, heavy smoking (≥ 10 cigarettes a day), and systemic diseases such as uncontrolled diabetes mellitus. All patients were healthy, with no disease that might affect the treatment outcome. The patients were fully informed about the procedures, including the surgery, graft materials, and dental implants, and written, informed consent was obtained from all of them. The research protocol was approved by the Ethics Committee of Dokkyo Medical University School of Medicine (R-10-14).

### HPMM β-TCP

**Fig. 1:**
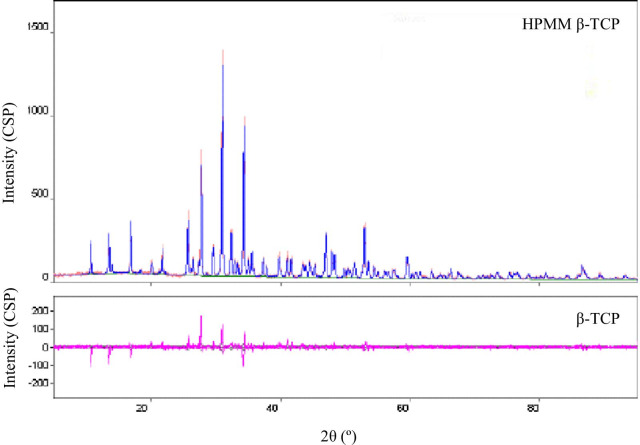
Analysis of X-ray diffraction of HPMM β-TCP. The synthesized granules consisting of pure-phase beta-tricalcium phosphate are recognized as high purity (more than 95%) by X-ray diffraction analysis

**Fig. 2:**
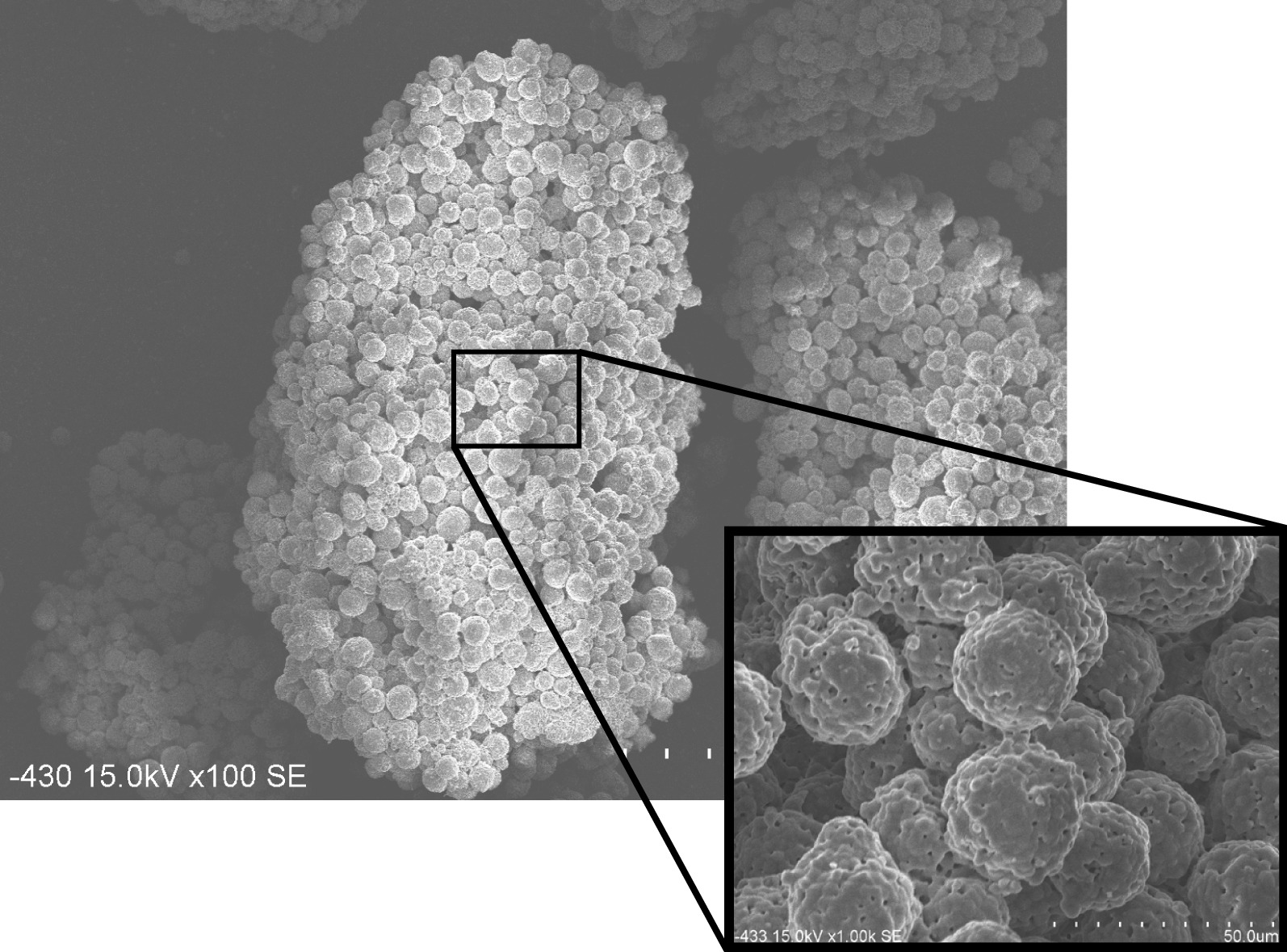
SEM micrograph of HPMM β-TCP. HPMM β-TCP is a granular bone substitute having a macro/microporous structure

HPMM β-TCP (Arrowbone-β-dental®) is a monophase β-TCP ceramic generated by the spray-dry method. The synthesized granules consisting of pure-phase beta-tricalcium phosphate are recognized as high purity (more than 95%) by X-ray diffraction analysis (Fig. 1). HPMM β-TCP is a granular bone substitute having a macro/microporous structure (Fig. 2) [23].

Maxillary sinus floor elevation procedure, dental implant placement, and bone biopsy retrieval.

**Fig. 3:**
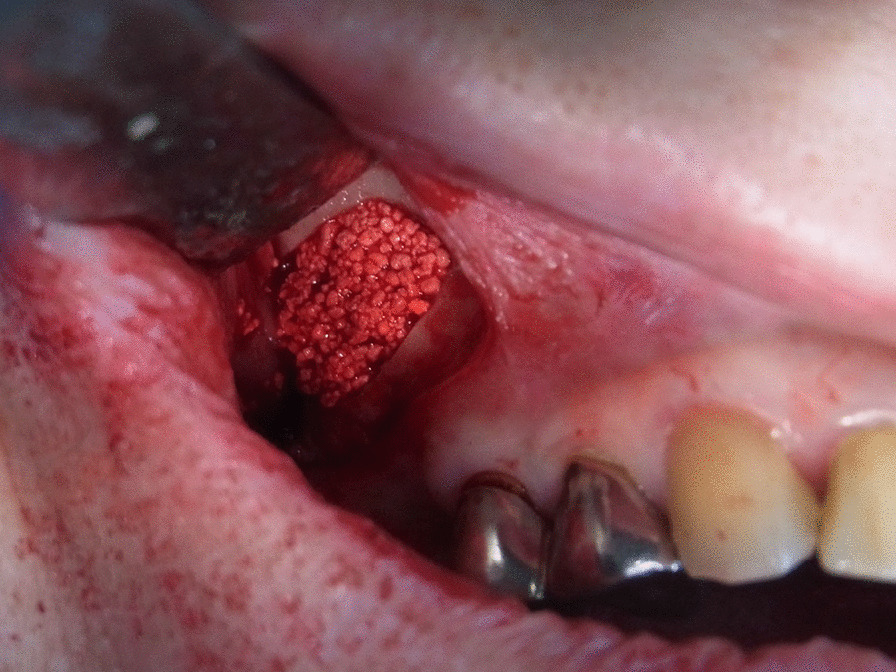
Use of HPMM β-TCP with a resorbable collagen membrane in sinus floor elevation. In all cases, the sinus membrane is covered with a resorbable collagen membrane (Bio-Gide®, Geistlich Pharma AG, Wolhusen, Switzerland). The space between the lifted sinus membrane and the sinus floor is filled with HPMM β-TCP

For unilateral maxillary sinus floor elevation, 9 patients underwent an outpatient procedure under local anesthesia, and 1 patient was treated under local anesthesia with intravenous sedation. An antibiotic (amoxicillin 1000 mg daily in four divided doses a day) for 7 days, and an oral rinse, ConCoolF (Weltec Corporation, Osaka, Japan) formulated with 0.0006% chlorhexidine digluconate, 0.0016% ethanol, and green tea extract (25 ml for 1 min, three times a day [25] for 2 weeks were prescribed for all patients. Via paracrestal incision and elevation of a mucoperiosteal flap, the maxillary wall was prepared. The lateral maxillary sinus wall was prepared using a round burr with irrigation with sterile saline. A buccal window together with the carefully elevated sinus membrane was dissected and reflected inward. The sinus membrane was detached from the maxillary walls and moved in the cranial direction. Visible small perforations of the sinus membrane were observed in 4 patients. In all cases, the sinus membrane was covered with a resorbable collagen membrane (Bio-Gide®, Geistlich Pharma AG, Wolhusen, Switzerland). The space between the lifted sinus membrane and the sinus floor was filled with HPMM β-TCP (Fig. 3). Complete wound closure was performed with 4/0 monofilament sutures. Sutures were removed two weeks later. After an approximately 7-month healing period (226.7 ± 12.3 days), elevation of a mucoperiosteal flap was performed for dental implant placement under local anesthesia in all cases. Before dental implant preparations were made, one bone biopsy was taken using a trephine drill with an external diameter of 3.3 mm from the grafted area at the one position planned dental implant insertion each patient, with sterile saline irrigation. We placed one implant body or two implant bodies on the grafted area in each patient. Total 16 dental implant bodies were placed in this experiment, 7 implant bodies with a length of 10 mm and diameter of 4.0 mm and 9 implant bodies with a length of 12 mm and diameter of 4.0 mm. All surgical procedures were performed by one oral surgeon (AF). Attention was paid to prevent premature loading of the dental implants. After a healing period of 6 months, the implants were loaded.

### Morphometric evaluation by radiological examination

**Fig. 4:**
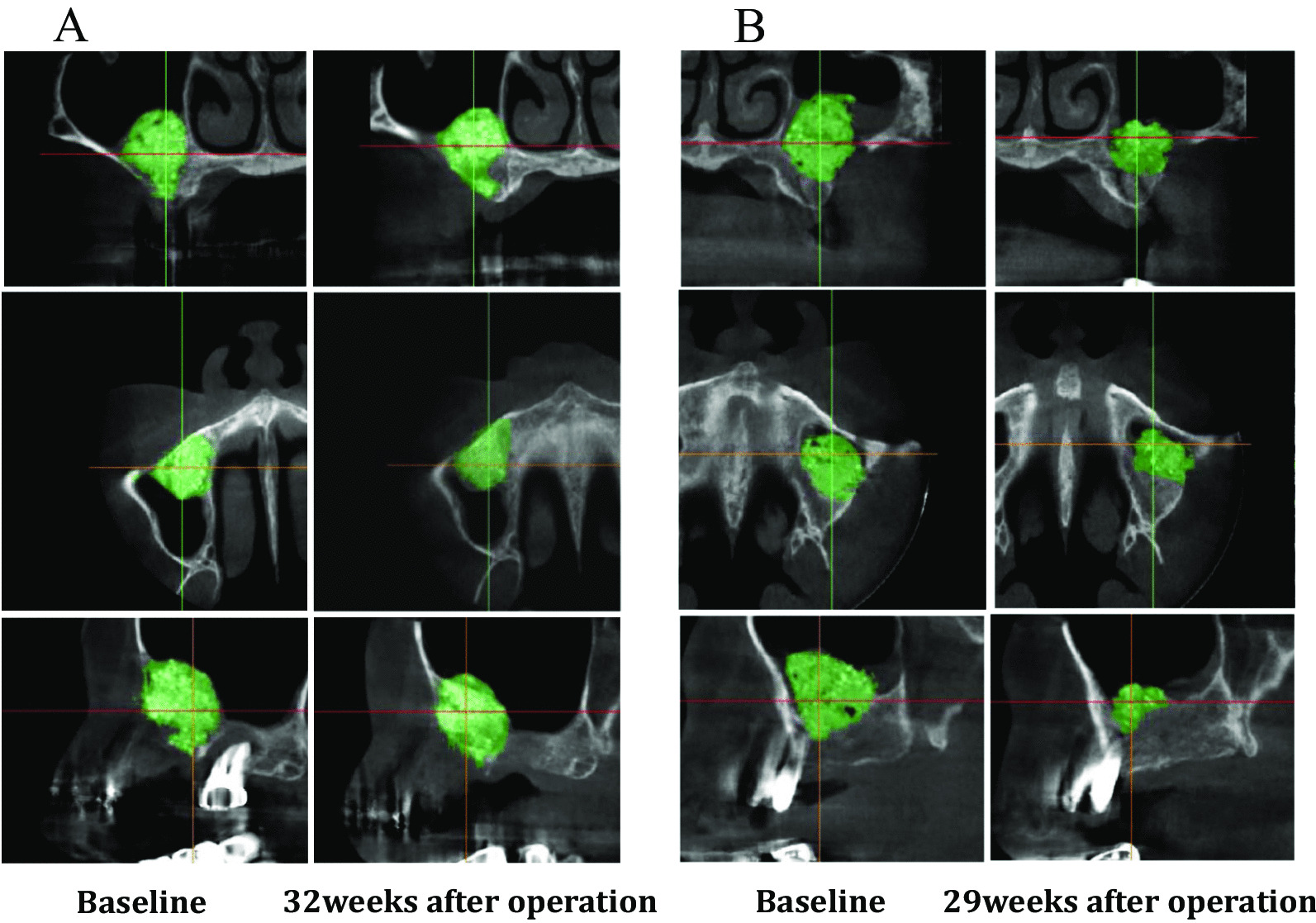
Analysis of the height and volume of the augmented bone using the reformatting imaging software. There are no differences in the height and the volume between Baseline and 32 weeks after Baseline in Case 1, whereas there are large differences in the height and volume between Baseline and 32 weeks after Baseline in Case 2

Panoramic radiographs and CT images were taken before surgery, after the maxillary sinus floor elevation procedure (Baseline), and prior to dental implant placement (approximately 7 months after the maxillary sinus floor elevation). CT images were stored in DICOM format and analyzed in SIMPLANT Pro 16 (Dentspy Sirona Inc., New York, NY, USA), a reformatting imaging software package that simulates the placement of dental implants. Images were analyzed by an independent experienced dentist without any clinical information (HK). The region of interest (ROI) was manually sampled in the site where the HPMMβ-TCP granules were embedded, and the HPMMβ-TCP granules were clearly visualized by the volume rendering method along the ROI. The boundaries were determined by reading each slice of the horizontal, coronal, and sagittal sections if the boundary between HPMMβ-TCP granules and the residual surrounding bone became unclear. For the height of the maxillary grafted materials or the mixture of both the maxillary grafted materials and the newly formed bone after the maxillary sinus floor elevation, the maximum height of the maxillary grafted materials or the mixture of both the maxillary grafted materials and the newly formed bone was measured from the top to the bottom for each case on the horizontal, coronal, and sagittal sections. For the volume of the maxillary grafted materials or the mixture of both the maxillary grafted materials and the newly formed bone after the maxillary sinus floor elevation, the volume of the maxillary grafted materials or the mixture of both the maxillary grafted materials and the newly formed bone drawn by the volume rendering method was calculated (Fig. 4) [26, 27].

### Histopathological and histomorphometric evaluation by bone biopsy

The specimens were placed in 4% formalin solution for 72 h and then in a 6% nitric acid solution for 12 h. The specimens were washed for 4 h and subsequently dehydrated in an ascending series of alcohol and xylene. Bone cores were finally embedded in paraffin. Fine-cut, 5-µm-thick sections were made from each specimen parallel to the long axis of the cylindrical core using a microtome. Subsequent to deparaffinization, the sections were exposed to a descending series of alcohol and xylene, then washed with water. Finally, the specimens were stained with hematoxylin and eosin.

**Fig. 5:**
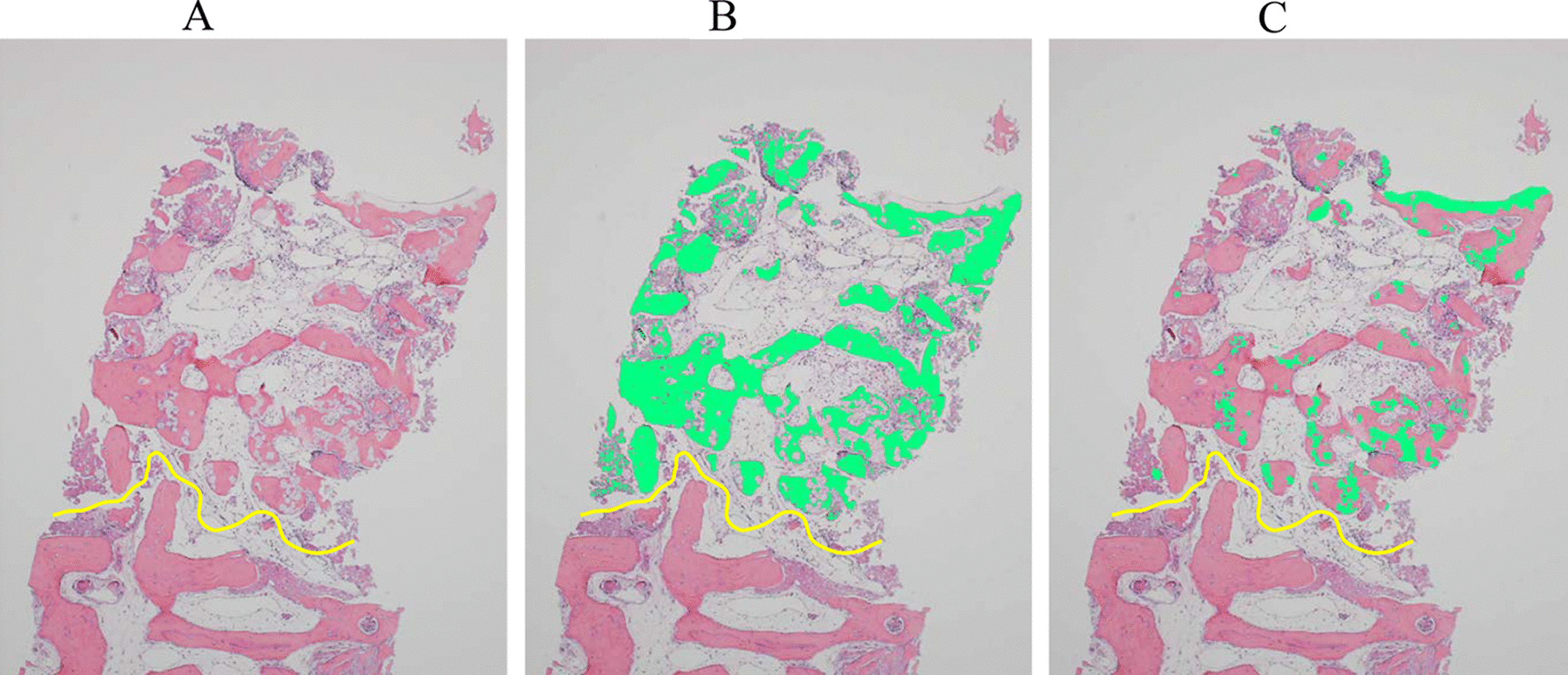
Overview of a typical example of a bone biopsy. **A** Newly formed bone area and native alveolar bone area divided by the yellow line. **B** Newly formed bone area filled by yellowish green. **C** Area of residual graft particles filled by yellowish green

Images of the sections were obtained with a digital camera (Olympus DP 80, Olympus, Tokyo, Japan) attached to a microscope (Olympus BX53, Olympus). The obtained images were transferred to a computer, and image analysis software (WinROOF2015 Standard®, Mitani Co., Fukui, Japan) was used for histomorphometric evaluations. Histomorphometric evaluations were performed over the total section of the biopsy, including residual and newly formed bone. Newly formed bone of the biopsy was defined as the area from the first visible HPMM β-TCP particle at the cranial side of the native alveolar bone (Fig. 5) [28]. The following histomorphometric measurements were performed: (1) new bone formation in the grafted area (percentage of newly formed bone area to total measured area); (2) area of graft particles (percentage of graft particle area to total measured area); and (3) soft-tissue area in the grafted zone (percentage of soft-tissue area to total measured area).

### Statistical analysis

The results of the morphometric analyses are shown as the mean ± SEM for each variable. The significance of differences was tested by the Wilcoxon signed-rank sum test. P values < 0.05 were considered significant. Effect size was calculated by γ (≥ 0.1: small, ≥ 0.3: medium, ≥ 0.5: large) for the Wilcoxon signed-rank test.

## Results

### Morphometric evaluation

No surgical site infections were observed following all surgical procedures. All the dental implants were osseointegrated after the 6 months healing period, then all the dental implants with the prosthesis were clinically functional after appropriate occlusal load.

**Fig. 6:**
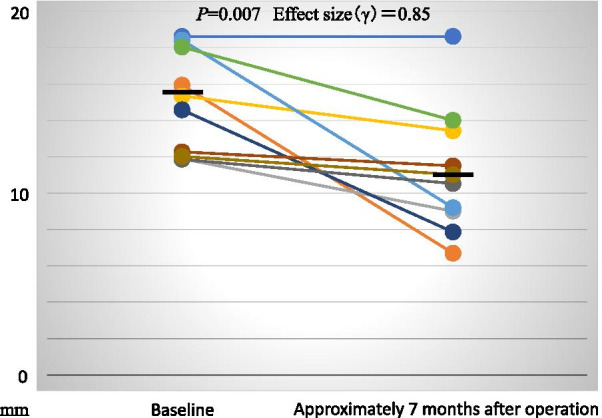
The height of the maxillary grafted materials. The heights of the mixture of both the maxillary grafted materials and the newly formed bone at approximately 7 months after sinus floor elevation are significantly lower than those of the maxillary grafted materials at Baseline (*P* = 0.007, Effect size (γ) = 0.85)

CT images were evaluated to assess the maximum height of the maxillary grafted materials at Baseline, as well as those of the mixture of both the maxillary grafted materials and the newly formed bone at approximately 7 months after the maxillary sinus floor elevation. The average height of the maxillary grafted materials at Baseline was 15.60 ± 0.88 mm. The average height of the mixture of both the maxillary grafted materials and the newly formed bone at approximately 7 months after the maxillary sinus floor elevation was 11.27 ± 1.09 mm. The rate of change from the average height of the maxillary grafted materials at Baseline to those of the mixture of both the maxillary grafted materials and the newly formed bone at approximately 7 months after the maxillary sinus floor elevation was 72.61% ± 6.44%. The heights of the mixture of both the maxillary grafted materials and the newly formed bone at approximately 7 months after sinus floor elevation were significantly lower than those of the maxillary grafted materials at Baseline (*P* = 0.007, Effect size (γ) = 0.85, Fig. 6).

**Fig. 7:**
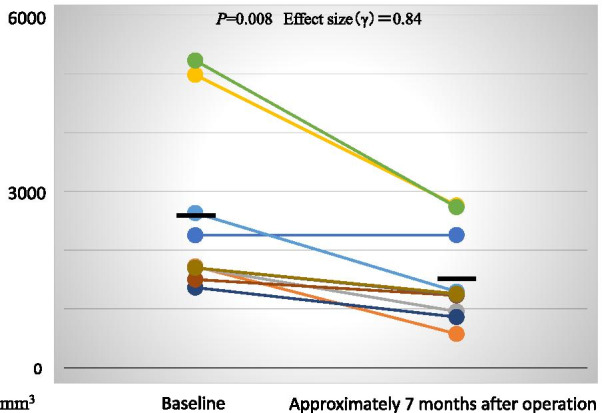
The volume of the maxillary grafted materials. The volumes of the mixture of both the maxillary grafted materials and the newly formed bone at approximately 7 months after the maxillary sinus floor elevation are significantly lower than those of the maxillary grafted materials at approximately 7 months after sinus floor elevation (*P* = 0.007, Effect size (γ) = 0.85). The volume prior to implant insertion is significantly lower than at Baseline

CT images were evaluated to assess the volume of the maxillary grafted materials at Baseline, as well as the volume of the mixture of both the maxillary grafted materials and the newly formed bone at approximately 7 months after the maxillary sinus floor elevation. The average volume of the maxillary grafted materials at Baseline was 2674.39 ± 453.34 mm^3^. The average volume of the mixture of both the maxillary grafted materials and the newly formed bone at approximately 7 months after the maxillary sinus floor elevation was 1516.88 ± 246.71 mm^3^. The rate of change from the average volume of the maxillary grafted materials at Baseline to those of the mixture of both the maxillary grafted materials and the newly formed bone at approximately 7 months after the maxillary sinus floor elevation was 61.45% ± 6.01%. The volumes of the mixture of both the maxillary grafted materials and the newly formed bone at approximately 7 months after the maxillary sinus floor elevation were significantly lower than those of the maxillary grafted materials at approximately 7 months after sinus floor elevation (*P* = 0.008, Effect size (γ) = 0.84, Fig. 7).

### Histopathological evaluation

**Fig. 8:**
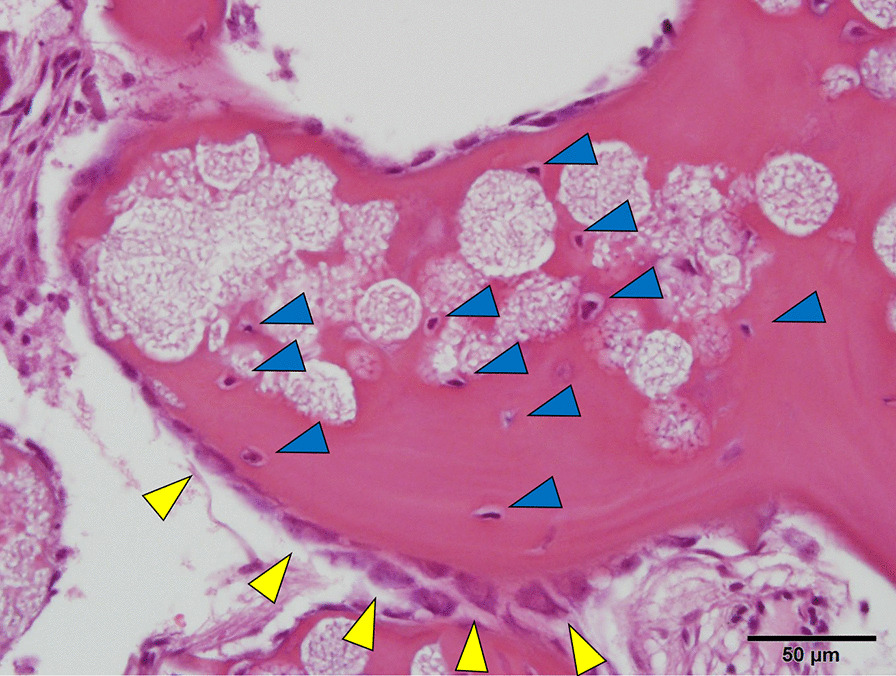
Histologic view of biopsy material in the newly formed bone area, hematoxylin and eosin staining. The new bone consisted of both lamellar and woven bone. Osteocytes were present in the lacunae. The HPMM β-TCP particles were integrated into the network of newly formed trabecular bone. Osteoblasts were detected next to the characteristic outlines of the newly formed bone. Yellow arrows: Osteoblasts. Blue arrows: Osteocytes

Histopathological examinations showed no infiltration of inflammatory cells in the tissue close to the grafted material. New bone formation was seen in all 10 specimens. The trabecular bone that formed from the residual bone expanded the grafted space. The new bone consisted of both lamellar and woven bone. Osteocytes were present in the lacunae. In 9 of 10 specimens, the primary trabecular structure was already reinforced by lamellar bone in some areas. The HPMM β-TCP particles were integrated into the network of newly formed trabecular bone. Close bone-to-substitute contact was observed. Osteoblasts were detected next to the characteristic outlines of the newly formed bone (Fig. 8).

### Histomorphometric evaluation

Table 1 shows the results of the histomorphometric measurements of HPMM β-TCP. Mean new bone formation was 33.97% ± 2.79%. The mean percentage of the residual grafted particle area was 15.81% ± 4.52%. The mean percentage of the soft-tissue area was 50.21% ± 4.28% (Table 1).

**Table 1:** Mean percentages of new bone, graft particles, and soft tissue areas (HPMM β-TCP)

Parameter	Mean	SEM
New bone (%)	33.97	2.79
Graft particles (%)	15.81	4.52
Soft tissue areas (%)	50.21	4.28

## Discussion

The maxillary sinus floor elevation procedures using HPMM β-TCP in this study showed uneventful healing. HPMM β-TCP, a new bone substitute, was thought to have good osteoconductive properties with proper absorbency characteristics. The planned amount of HPMMβ-TCP filled in the maxillary sinus bed for elevation should overcorrect to compensate for the expected resorption.

The limitation of this study is as follows: because this was a retrospective, observational study of the usage of HPMM β-TCP, but not an interventional study, the outcome of the procedure with HPMM β-TCP cannot be compared with the outcomes of procedures using other bone substitutes on the same patients undergoing maxillary sinus floor elevation. The purpose of this study was to analyze the effectiveness of a new bone substitute, HPMM β-TCP, approved worldwide as ordinary treatment for bone augmentation. Additional experiments might be necessary to confirm the current observations.

Tricalcium phosphate (TCP) is biocompatible, osteoconductive calcium phosphate. The biomaterial can provide a scaffold for potential bony ingrowth [29]. TCP resorbs, unlike BHA, and it is replaced by newly formed bone [21, 22]. The problem is that this replacement does not necessarily occur in a 1:1 ratio. Often less bone is produced as compared with the volume of TCP resorbed [14, 15, 30]. Several authors have reported that β-TCP is a satisfactory graft material for augmentation of the maxillary sinus [11–16, 19–22]. Wiltfang et al. reported the degradation characteristics of β-TCP in minipigs. In seven Goettingen minipigs, cancellous bone defects of 3.5–4.7 ml were created in the area of the tibial head, and the defects were filled with β-TCP. After 86 weeks, approximately 3% β-TCP was still found. These residual particles stay within the newly formed trabeculae, which show a functional orientation. Following absorption and bony substitution, β-TCP ceramics allowed a restitutio ad integrum at the grafted bone tissue [29].

Zijderveld et al. described maxillary sinus floor augmentation with β-TCP (Cerasorb®; Curasan, Kleinostheim, Germany) using the same method as the present study [15]. They reported mean new bone formation of 17% ± 5%, and the mean percentage of residual graft particle area 6 months postoperatively was 31% ± 4% [15]. The present study showed that HPMM β-TCP has a good balance with bone formation and material absorption. The reason for these results was likely both the high purity and the macro/microporous structure of HPMM β-TCP. HPMM β-TCP has a surface area of 0. 352 m2/g, while Ceresorb® has a surface area of 0.17 m2/g 23. This indicates that HPMM β-TCP has a higher porosity than Ceresorb®. The greater volume of new bone formation using 100% HPMM β-TCP was confirmed with lower percentages of residual graft particles in comparison with β-TCPs [15, 30–32] (Table 2).

**Table 2:** Comparison of the β-TCPs and HPMM β-TCP

Bone substitute	New bone (%)	Graft particles (%)
β-TCP A (Cerasorb® 15)	17.5 ± 5	31 ± 4
β-TCP B (Suprabone® 30)	33.40 ± 10.43	30.39 ± 10.29
β-TCP C (Bioresorb® 31)	36.16 ± 12.61	30.26 ± 11.70
β-TCP D (kasios® 32)	21.09 ± 2.86	34.05 ± 3.01
HPMM β-TCP (Arrowbone-β- dental®)	33.97 ± 7.98 (SDs)	15.81 ± 14.78 (SDs)

Histologically, the vital bone was composed of both lamellar and woven bone. Osteocytes were present in the lacunae. The newly formed bone was in intimate contact with the substitute material, outlining the osteoconductive properties of the HPMM β-TCP material. Bone maturation was evident with the presence of lamellar bone. Osteoblasts were detected next to the characteristic outlines of the newly formed bone (Fig. 8). Retrograde infection with viruses and bacteria via the ostomeatal complex from the nasal cavity causes acute paranasal sinusitis. If there is no activity in the bone of the maxillary sinus, paranasal sinusitis will get worse, and the inactive bone of the maxilla has no potential as supporting tissue for dental implants. Therefore, bone activity is important for the alveolar part of the posterior maxilla for dental implants.

BHA is a deproteinized natural bovine cancellous bone with a crystalline structure that is very similar to human bone. The deproteinization process through chemical and heat treatments should eliminate any immunogenicity concerns. However, Schwartz et al. suggested that BHA can contain osteoinductive proteins, such as transforming growth factor-β, bone morphogenic protein, and bone morphogenic protein-2, which can elicit an immune response [33]. One case report presented a foreign body reaction in a patient who had undergone alveolar ridge augmentation with a mixture of BHA and autogenous bone [34]. The most plausible explanation for such a reaction is an interaction between residual proteins within BHA particles with adhesion receptors present on monocyte/macrophage inflammatory cell populations. Scolozzi et al. reported that fungus ball formation was frequently observed in the maxillary sinus augmented by BHA [35].

Taking the present study together with previous reports, we recommend a protein-free bone-grafting material with less residual substitute after bone formation and with good osteoconductive properties, such as HPMM β-TCP, for maxillary sinus floor elevation. It is the limitation of this study that use of HPMM β-TCP could not be compared with use of other bone substitutes in the same patients who underwent maxillary sinus floor elevation or other patients in the same surgical period. The purpose of this study was to analyze the effectiveness of a bone substitute approved worldwide in the ordinary treatment for bone augmentation. Additional experiments, such as prospective cohort study and randomized controlled trial, might be necessary to confirm the current observations in the future.

## Conclusions

On histological and histomorphometric evaluations, HPMM β-TCP, a new bone substitute, was thought to be a suitable material with good osteoconductive properties and proper absorbency characteristics for maxillary sinus floor elevation.

## Data Availability

The datasets generated and analyzed during the current study are available from the corresponding author upon reasonable request.

## Abbreviations

CT: Computed tomography
HPMM β-TCP: High-purity macro/microporous beta-tricalcium phosphate
BHA: Bovine-derived hydroxyapatite
β-TCP: Beta-tricalcium phosphate
TCP: Tricalcium phosphate
